# Fatigue sensation induced by the sounds associated with mental fatigue and its related neural activities: revealed by magnetoencephalography

**DOI:** 10.1186/1744-9081-9-24

**Published:** 2013-06-13

**Authors:** Akira Ishii, Masaaki Tanaka, Masayoshi Iwamae, Chongsoo Kim, Emi Yamano, Yasuyoshi Watanabe

**Affiliations:** 1 Department of Physiology, Osaka City University Graduate School of Medicine, 1-4-3 Asahimachi, Abeno-ku, Osaka, 545-8585, Japan; 2 RIKEN, Center for Molecular Imaging Science, 6-7-3 Minatojima-minamimachi, Chuo-ku, Hyogo, 650-0047, Japan

**Keywords:** Fatigue sensation, Chronic fatigue syndrome, Classical conditioning, Magnetoencephalography (MEG), Equivalent current dipole (ECD)

## Abstract

**Background:**

It has been proposed that an inappropriately conditioned fatigue sensation could be one cause of chronic fatigue. Although classical conditioning of the fatigue sensation has been reported in rats, there have been no reports in humans. Our aim was to examine whether classical conditioning of the mental fatigue sensation can take place in humans and to clarify the neural mechanisms of fatigue sensation using magnetoencephalography (MEG).

**Methods:**

Ten and 9 healthy volunteers participated in a conditioning and a control experiment, respectively. In the conditioning experiment, we used metronome sounds as conditioned stimuli and two-back task trials as unconditioned stimuli to cause fatigue sensation. Participants underwent MEG measurement while listening to the metronome sounds for 6 min. Thereafter, fatigue-inducing mental task trials (two-back task trials), which are demanding working-memory task trials, were performed for 60 min; metronome sounds were started 30 min after the start of the task trials (conditioning session). The next day, neural activities while listening to the metronome for 6 min were measured. Levels of fatigue sensation were also assessed using a visual analogue scale. In the control experiment, participants listened to the metronome on the first and second days, but they did not perform conditioning session. MEG was not recorded in the control experiment.

**Results:**

The level of fatigue sensation caused by listening to the metronome on the second day was significantly higher relative to that on the first day only when participants performed the conditioning session on the first day. Equivalent current dipoles (ECDs) in the insular cortex, with mean latencies of approximately 190 ms, were observed in six of eight participants after the conditioning session, although ECDs were not identified in any participant before the conditioning session.

**Conclusions:**

We demonstrated that the metronome sounds can cause mental fatigue sensation as a result of repeated pairings of the sounds with mental fatigue and that the insular cortex is involved in the neural substrates of this phenomenon.

## Background

Fatigue sensation plays an important role as a biological alarm under the conditions of fatigue and urges us to take a rest to avoid upsetting homeostasis and to recover from fatigue. In this sense, the fatigue sensation is beneficial for our survival. However, over-activation of the fatigue sensation might be involved in the pathophysiology of fatigue-related diseases, such as chronic fatigue syndrome [1]. The hypothesis, named the “Co-conditioning Theory of Fatigue,” has been proposed as an etiology of chronic fatigue syndrome, in which classical conditioning of the fatigue sensation plays an important role [1]. According to the co-conditioning theory, even expectations of overwork and/or stress without impairment of homeostasis (conditioned stimulus) cause severe fatigue sensations, as a result of repeated pairings of impaired homeostasis and function (unconditioned stimulus) with expectations of overwork and/or stress. Therefore, it is important to determine whether the classical conditioning of fatigue sensation can take place and to clarify the neural mechanism involved in the classical conditioning of fatigue sensation. In this study, we focused on mental fatigue induced by demanding mental task trials, since many people suffer from this type of fatigue in our modern society.

Classical conditioning of the fatigue sensation has been reported in an animal model of fatigue [2]. In that study, rats received paired conditioned and unconditioned stimuli. Feeding of a sucrose solution was used as a conditioned stimulus, and intraperitoneal injection of a synthetic double-stranded RNA, polyriboinosinic:polyribocytidylic acid (poly I:C), was used as a unconditioned stimulus. Fatigue is defined as difficulties in initiating or sustaining voluntary activities [3]. Because the poly I:C injection has been shown to be related to decreased spontaneous activity on the running wheel, injection of the poly I:C has was used to make an animal model of fatigue [4-6]. After a 4-day conditioning period, the rats conditioned with the feeding of the sucrose solution showed decreased spontaneous activity when given only the sucrose solution.

Although there has been no reports on classical conditioning of the fatigue sensation in humans, it has been reported that classical conditioning is involved in the pathophysiology of certain human diseases. For example, the involvement of classical conditioning in the mechanism of pain, another biological alarm, has been reported in humans [7-9]. In fact, classical conditioning is an important modulator of chronic pain [10].

The aim of our study was to examine whether classical conditioning of the fatigue sensation can take place in humans and to identify the neural substrates of the fatigue sensation related to classical conditioning. To this end, we tried to classically condition human participants to experience a mental fatigue sensation when they were listening to sounds, using fatigue-inducing mental task trials (two-back task trials) as the unconditioned stimuli and metronome sounds as the conditioned stimuli (conditioning group). Two-back task is a demanding working-memory task in which participants have to retain the letters appeared on the display and judge whether the letter presented at the moment was the same as the one that had appeared two presentations before. It has been reported that performing two-back task trials for 30 min induces mental fatigue [11,12]. We then compared neural activities between the unconditioned and conditioned states to identify the neural substrates of the mental fatigue sensation related to classical conditioning. In addition to the conditioning group, we performed additional experiment to confirm that repetitive listening to metronome sounds did not induce the fatigue sensation (control group). We used magnetoencephalography (MEG) with high temporal resolution to measure neural activities related to classical conditioning of the fatigue sensation, since the neural correlates may include several brain regions and temporal sequences among these brain regions may provide important clues to clarify the neural mechanisms of classical conditioning of the fatigue sensation. To assess the level of fatigue induced by the metronome sounds, we recorded electrocardiography (ECG) during the MEG measurements and performed frequency domain analysis of the R-R wave intervals to obtain low-frequency (LF) power and high-frequency (HF) power. Since it has been reported that increases in the LF/HF ratio and decreases in HF power were associated with mental fatigue [11,13], increased LF/HF ratio and reduced HF power seem to be a characteristic feature of mental fatigue.

## Methods

### Participants

Ten healthy male volunteers (aged 21.1 ± 0.3 years [mean ± SEM]) participated in conditioning group and 9 healthy volunteers (aged 36.6 ± 2.4 years; 5 male and 4 female) participated in control group. Current smokers, participants with a history of mental or brain disorder, or those taking chronic medications that affect the central nervous system were excluded. All participants provided written informed consent before participation. The study was approved by the Ethics Committee of Osaka City University and was conducted in accordance with the principles of the Declaration of Helsinki.

### Experimental design

#### Conditioning group

**Figure 1:**
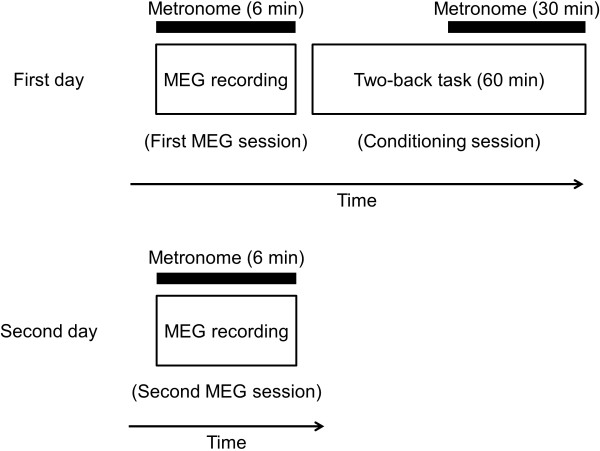
**Experimental design.** On the first day, neural activities during listening to metronome sounds for 6 min were measured using magnetoencephalography (MEG) (first MEG session). Thereafter, two-back task trials were performed for 60 min (conditioning session), in which metronome sounds were started 30 min after the start of the task trials, so that the fatigue sensation was conditioned against the metronome sounds (conditioned stimuli). On the second day, neural activities while listening to the metronome sound for 6 min were measured using MEG to evaluate the effect of conditioning of the fatigue sensation acquired on the first day on neural responses (second MEG session). The metronome sounds were same across all sessions.

Conditioning experiment consisted of two MEG recording sessions and one conditioning session. Participants joined the experiment on two successive days (Figure 1). We used metronome sounds as the conditioned stimuli. On the first day, neural activities while listening to metronome sounds with their eyes closed for 6 min were measured using MEG (first MEG session). Thereafter, fatigue-inducing mental task trials for 60 min were performed without MEG measurement (conditioning session). During these task trials, metronome sounds were started 30 min after the start of the mental task so that the fatigue sensation could be conditioned against the metronome sounds. On the second day, neural activities while listening to metronome sounds with their eyes closed for 6 min were measured again using MEG (second MEG session) to examine the effect of the conditioning session on neural responses. MEG measurements were performed in a magnetically shielded room at Osaka City University Hospital with the participants in a supine position.

#### Control group

We performed control experiment to confirm that repetitive listening to metronome sounds did not induce the fatigue sensation. Participants in the control group listened to the metronome sounds for six min (first metronome session) but did not perform conditioning session on the first day and listened to the sounds again on the second day (second metronome session). The metronome sounds were the same as that used in the MEG sessions and the conditioning session of the conditioning group. On the first and second days, they were asked to rate their subjective levels of mental fatigue on the VAS, immediately before and after they listened to the metronome sounds. They were asked to rate the levels of the fatigue sensation that was caused by listening to the metronome sounds on the VAS immediately after they listened to the metronome sounds on each day.

### Conditioning session

We used two-back task trials to induce the fatigue sensation and metronome sounds as the conditioned stimuli. During the two-back task trials, one of four letters was presented on a display of a personal computer every 3s and the participants had to judge whether the letter presented on the display at the moment was the same as the one that had appeared two presentations before. If it was, they were to press the right button with their right middle finger; if it was not, they were to press the left button with their right index finger. They were instructed to perform the task trials as quickly and as correctly as possible, and had to engage in two-back task trials for 60 min. Since it has been reported that performing two-back task trials for 30 min induces mental fatigue and mental fatigue sensation [11,12], i.e., it takes 30 min until fatigue and fatigue sensation develop, metronome sounds (every 2s) were started 30 min after the start of the task trials, and the sounds were continued until the end of the task. They were not informed about the metronome sounds before.

### Metronome sounds

The metronome sound was created using a freeware program (Art Metronome; Mu Tech, Nara, Japan; http://mu-tech.co.jp/Japanese/top.htm). The temporal duration of the sound was approximately 600 ms.

### Subjective level of mental fatigue

The participants were asked to rate their subjective levels of mental fatigue on a visual analogue scale (VAS) from 0 (minimum) to 100 (maximum), immediately before and after the MEG, conditioning, and metronome sessions. After the MEG and metronome sessions, they were asked to rate the levels of the fatigue sensation that was caused by listening to the metronome sounds on VAS. After the two-back task trials, they were also asked to rate how much they noticed the metronome sounds during the mental fatigue-inducing task trials on the VAS (hereinafter referred to as the notice level of metronome sounds).

### Magnetoencephalography session

We measured neural activities when the participants were listening to the metronome sounds. The MEG recordings on the first day and the second day were conducted in the same manner.

MEG recordings were performed using a 160-channel whole-head type MEG system (MEG vision; Yokogawa Electric Corporation, Tokyo, Japan) with a magnetic field resolution of 4 fT/Hz^1/2^ in the white-noise region. The sensor and reference coils were gradiometers 15.5 mm in diameter and 50 mm in baseline, and each pair of sensor coils was separated at a distance of 23 mm. The sampling rate was 1000 Hz with a 0.3-Hz high-pass filter.

### Magnetoencephalography analyses

Before processing the MEG data, magnetic noise originating from outside the shield room was eliminated by subtracting the data obtained from reference coils using a software program (MEG 160; Yokogawa Electric Corporation). The MEG data acquired on the first and second days were separately averaged offline after analogue-to-digital conversion with a band-pass filter of 3 to 30 Hz. The onset of each metronome sound was used as a trigger. The mean magnetic signal in the pre-stimulus time period (from 0 to 300 ms before the trigger) was subtracted from each channel to remove the baseline shift of MEG data. Epochs of the raw MEG data including artifacts were excluded from the analysis by careful visual detection of the artifacts before averaging. To identify the sources of the evoked activities, equivalent current dipole (ECD) analyses were performed using software (MEG 160; Yokogawa Electric Corporation). The ECDs with goodness of fit (GOF) values above 85%, that is, ECDs explaining > 85% of the field variance, were used, based on a previous report [14].

### Magnetic resonance imaging overlay

Anatomical magnetic resonance imaging (MRI) was performed on all participants using a Philips Achieva 3.0TX (Royal Philips Electronics, Eindhoven, The Netherlands) to permit registration of magnetic source locations with their respective anatomic locations. Before MRI scanning, five adhesive markers (Medtronic Surgical Navigation Technologies Inc., Broomfield, CO) were attached to the skin of the head (the first and second ones were located 10 mm in front of the left tragus and right tragus, the third at 35 mm above the nasion, and the fourth and fifth at 40 mm right and left of the third one). MEG data were superimposed on MR images using information obtained from these markers and MEG localization coils.

### Transforming MEG source locations to Montreal Neurological Institutes (MNI) coordinates

The ECD locations and orientations expressed in the participant-specific head coordinate system were transformed into common MNI coordinates. The structural MRI was normalized with the MNI template using statistical parametric mapping 8 (SPM8; The Welcome Trust Centre for Neuroimaging, London, UK; http://www.fil.ion.ucl.ac.uk/spm) implemented in MATLAB 2010b (The MathWorks, Natic, MA), and 12 parameters were obtained from linear transformation (i.e., affine transformation). Then ECD locations and orientations were transformed based on the linear transformation using these 12 parameters.

### Electrocardiography

To examine changes of autonomic nerve activities caused by conditioning of the fatigue sensation, ECG was recorded during the MEG measurements. The ECG data were analyzed using MemCalc for Windows (Global Medical Solution Inc., Tokyo, Japan). R-R wave variability was measured as an indicator of autonomic activity. For frequency domain analysis of the R-R wave intervals, low-frequency (LF) power was calculated as the power within the frequency range of 0.04 to 0.15 Hz, and high-frequency (HF) power was calculated as that within the frequency range of 0.15 to 0.4 Hz. LF and HF were measured in absolute units (ms^2^). The HF is vagally mediated [15-17], but the LF originates from a variety of sympathetic and vagal mechanisms [15,18]. The LF/HF ratio is considered an index of sympathetic nervous system activity [19].

### Statistical analyses

Values are presented as mean ± SEM, unless otherwise stated. Two-way analysis of variance (ANOVA) for repeated measures were performed to assess the effect of the conditioning and the time course on the subjective level of mental fatigue and the indices of autonomic activities (LF, HF, and LF/HF ratio). The paired t-test was used to evaluate significant differences between the two conditions. Categorical variables (the number of participants in whom specific ECDs were observed in the first and second MEG sessions) were compared using the McNemar test to determine whether the proportion of participants in whom specific ECDs were observed only in the second MEG session was significantly higher than that in the first MEG session. The McNemar test is a statistical method used to determine whether the row and column marginal frequencies in a 2 × 2 contingency table are equal or unequal [20]. Pearson’s correlation analyses were used to evaluate the relationship between the level of fatigue sensation caused by conditioned stimuli and the notice level of conditioned stimuli. All P values were two-tailed, and values less than 0.05 were considered statistically significant. Statistical analyses were performed using the SPSS 18.0 software package (SPSS, Chicago, IL).

## Results

### VAS scores

#### Control group

The levels of subjective mental fatigue sensation before and after listening to the metronome sounds in the first day were 21.0 ± 3.8 and 23.3 ± 4.4, respectively. The levels of subjective mental fatigue sensation before and after listening to the metronome sounds in the second day were 18.1 ± 4.6 and 18.6 ± 5.0, respectively. The levels of subjective fatigue sensation caused by listening to the metronome sounds in the first day and second day were 7.3 ± 4.8 and 6.7 ± 5.0, respectively. The level of subjective fatigue sensation caused by listening to the metronome sounds in the second day was not altered compared to that in the first day (P = 0.332, paired t-test).

#### Conditioning group

**Figure 2:**
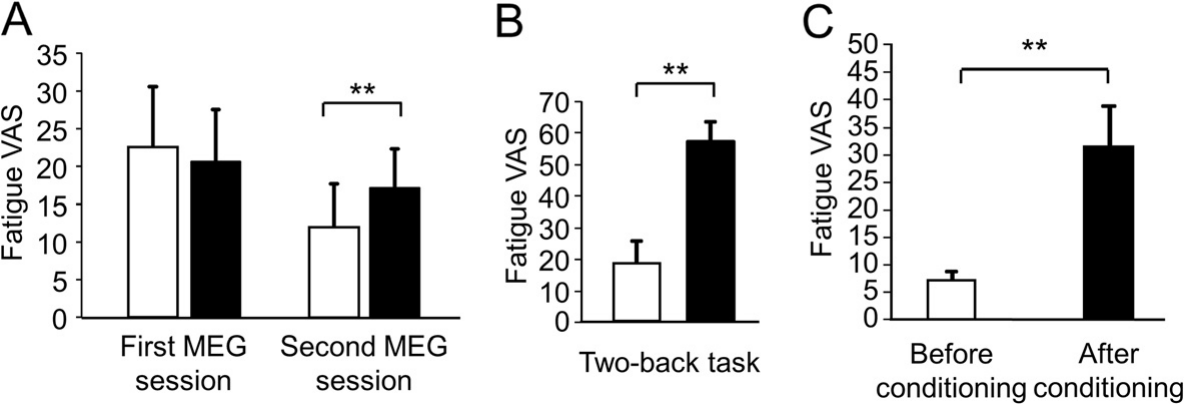
**Visual analog scale (VAS) for fatigue.** (**A**) Subjective levels of mental fatigue immediately before (open columns) and after (closed columns) first and second magnetoencephalography (MEG) sessions. (**B**) Subjective levels of mental fatigue immediately before (open column) and after (closed column) 60-min two-back task session. (**C**) Subjective levels of mental fatigue caused by conditioned stimuli during the MEG recordings on the first day (open column) and on the second day (closed column). Data are presented as mean and SEM (n = 10). **P < 0.01 (paired t-test).

**Figure 3:**
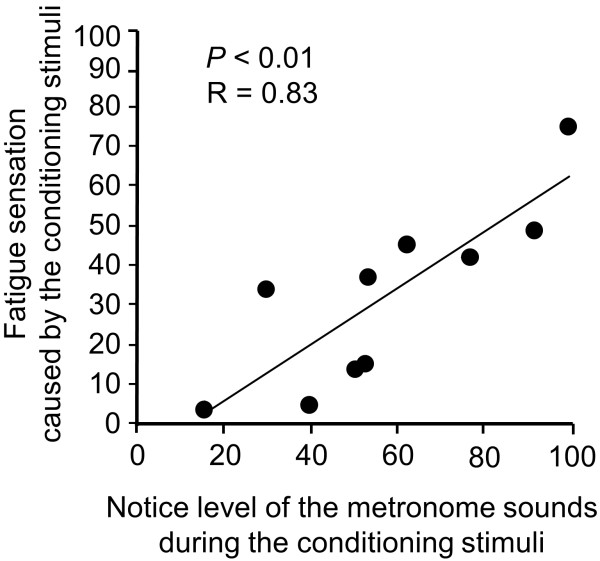
**Relationship between the level of fatigue sensation caused by the conditioned stimuli and the notice level of metronome sounds during the conditioned stimuli assessed using visual analogue scales.** The level of fatigue sensation induced by the conditioning stimuli was positively correlated with the notice level of the metronome sounds during the conditioned stimuli. Linear regression line, Pearson’s correlation coefficient (R), and P value are shown.

To assess changes in the level of subjective mental fatigue sensation before and after each MEG session, two-way ANOVA for repeated measures was performed. Results showed no significant main effects [main effect of conditioning, F(1, 9) = 0.83, P = 0.21; main effect of time course within the session, F(1, 9) = 1.52, P = 0.25]. However, results showed a conditioning × time course interaction effect [F(1, 9) = 7.18, P = 0.03]. The level of subjective mental fatigue sensation after the second MEG session was higher than that before the second MEG session (P < 0.01, paired t-test) (Figure 2A). The level of subjective mental fatigue sensation after the two-back task trials was higher relative to that before the task trials (P < 0.01, paired t-test) (Figure 2B). The level of subjective fatigue sensation caused by listening to the metronome sounds during the second MEG session was higher relative to that caused during the first MEG session (P < 0.01, paired t-test) (Figure 2C) and was positively correlated to the notice level of the metronome sounds during the conditioning session (Figure 3).

#### Two-way ANOVA for repeated measures to the control and conditioning groups

To assess the effect of listening to the metronome sounds during the conditioning session on the subjective level of fatigue sensation, two-way ANOVA for repeated measures to the control and conditioning groups was performed. Results showed significant main effect of time course (first day vs. second day) and conditioning × time course interaction effect [main effect of conditioning, F(1, 17) = 4.307, P = 0.053; main effect of time course, F(1, 17) = 10.317, P = 0.005; conditioning × time course interaction effect, F(1, 17) = 11.494, P = 0.003].

### Frequency analysis of ECG

**Figure 4:**
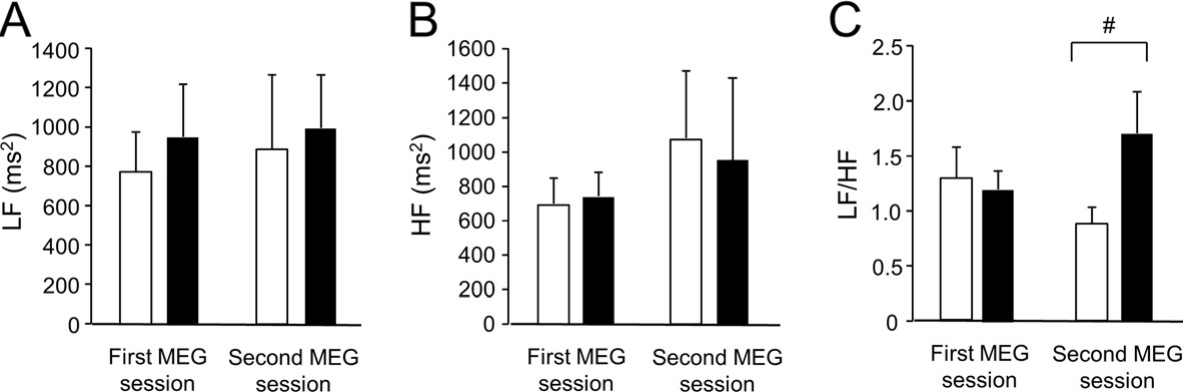
**Autonomic nerve activities assessed by the frequency analyses of electrocardiography.** Low-frequency power (**A**; LF), high-frequency power (**B**; HF), and LF/HF ratio (**C**) were assessed before (open columns) and after (closed columns) the first and second magnetoencephalography (MEG) sessions. Data are presented as means and SEM (n = 9). ^#^P < 0.10 (paired t-test).

The ECG data from one participant (participant No. 9) was excluded from the analyses because of an error in the data collection due to poor electrical contact of the ECG electrodes. To assess changes in LF, HF, and LF/HF ratio before and after each MEG session, two-way ANOVA for repeated measures were performed. LF [main effect of conditioning, F(1, 8) = 0.20, P = 0.67; main effect of time course within the session, F(1, 8) = 0.70, P = 0.43; conditioning × time course interaction effect, F(1, 8) = 0.19, P = 0.70] (Figure 4A), HF [main effect of conditioning, F(1, 8) = 0.63, P = 0.45; main effect of time course within the session, F(1, 8) = 0.16, P = 0.70; conditioning × time course interaction effect, F(1, 8) = 0.59, P = 0.46] (Figure 4B), and LF/HF ratio [main effect of conditioning, F(1, 8) = 0.04, P = 0.85; main effect of time course within the session, F(1, 8) = 2.28, P = 0.17; conditioning × time course interaction effect, F(1, 8) = 2.39, P = 0.16] (Figure 4C) showed no significant main effects or interaction effects. The LF/HF ratio after the second MEG session showed tendency toward increase compared to that before the session (P = 0.06, paired t-test) (Figure 4C).

### MEG data

**Figure 5:**
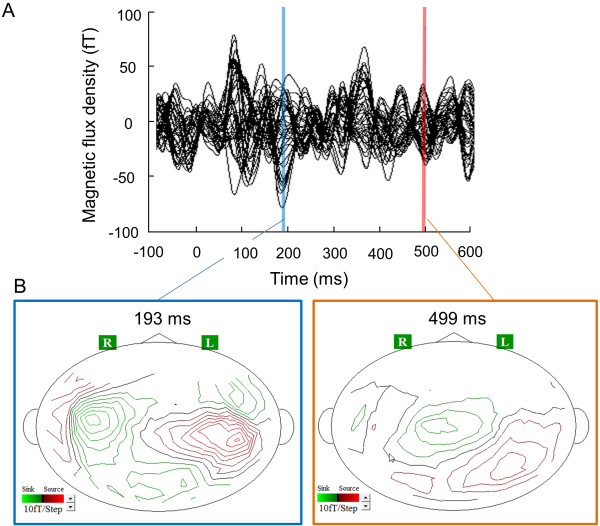
**Typical example of magnetic fields (A) and isofield contour maps (B) observed only in the second MEG session.** There were two major magnetic responses. The peak latencies after the onset of the sound were 193 ms and 499 ms. The right (R) and left (L) sides are indicated.

The MEG data for two participants were contaminated with magnetic noise caused by metal attached to the clothes in one participant (participant No. 7) and dental braces in another participant (participant No. 4). Thus, MEG data from these two participants were excluded from our analyses. A typical example (participant No. 1) of magnetic fields observed in the second MEG session is shown in Figure 5A. There were several magnetic responses both in the first and second MEG sessions including M100 responses produced by auditory stimuli. There were two major magnetic responses that were observed only in the second MEG session. The peak latencies of the first and second magnetic responses after the onset of each metronome sound were 193 ms and 499 ms, respectively. There were no corresponding magnetic responses observed in the first MEG session. Isofield contour maps corresponding to these two magnetic responses are shown in Figure 5B.

**Figure 6:**
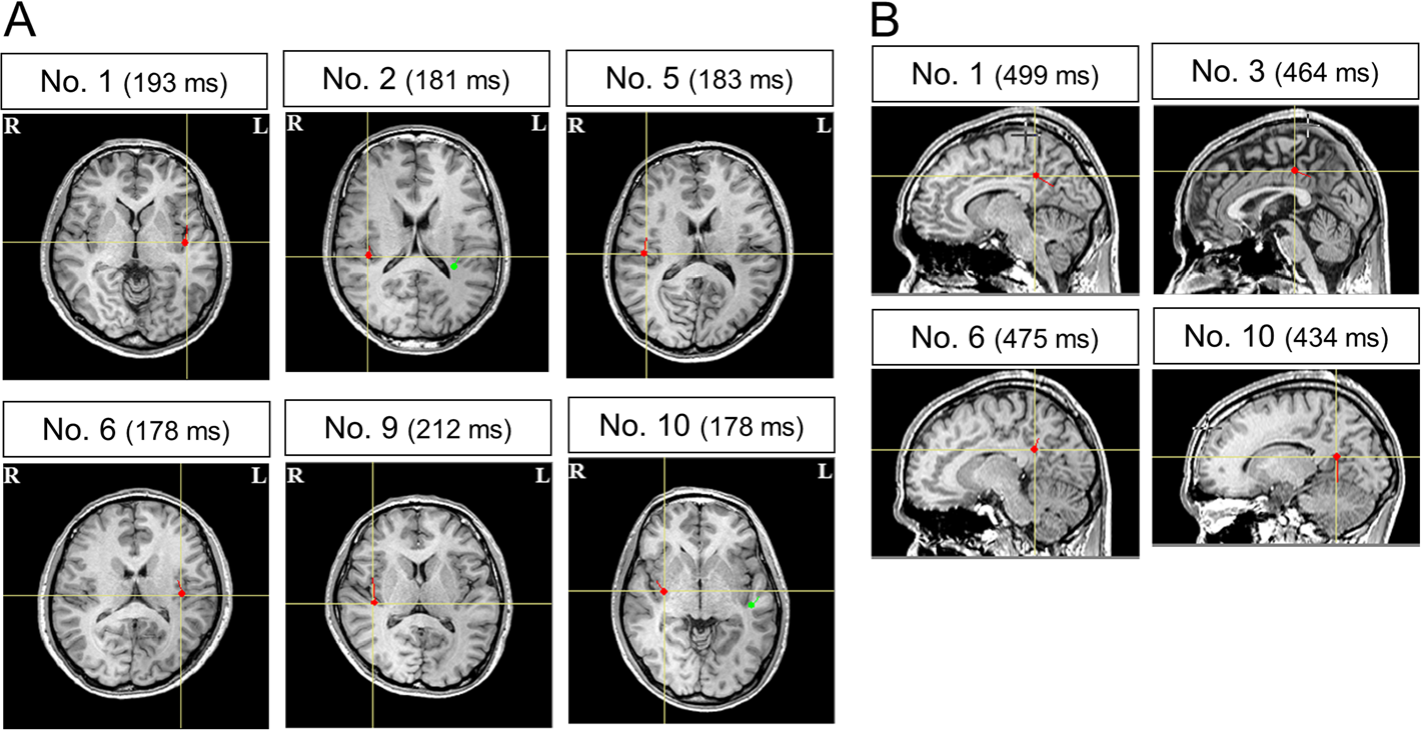
**Locations of the equivalent current dipoles (ECDs) induced by the conditioned stimuli.** (**A**) Magnetic responses with latencies approximately 190 ms were localized in the insular cingulate cortex in six participants (axial view) (R: right side; L: left side). Bilateral activations of the insular cortex were observed in participants No. 2 and No. 10. (**B**) Magnetic responses with latencies approximately 470 ms were localized in the posterior cingulate cortex in four participants (sagittal view). Closed red and green circles indicate the location of ECDs and short red and green lines radiating from closed circles indicate the orientation of ECDs. ECDs are superimposed on individual magnetic resonance images. The peak latencies are presented in parentheses.

In the ECD analyses, we could estimate the magnetic responses localized in the insular cortex in six of eight participants (two participants on the right side, two participants on the left side, and two participants on both sides), whose mean latencies were approximately 190 ms (Figure 6A), and the magnetic responses localized in the posterior cingulate cortex (PCC) in four of eight participants, whose mean latencies were approximately 470 ms (Figure 6B). The proportion of participants in whom ECDs in the insular cortex could be estimated in the second MEG session was statistically significant relative to that in the first MEG session (P < 0.05, McNemar test), although the proportion of participants in whom ECDs in the PCC could be estimated did not reach statistical significance (P = 0.13, McNemar test). The mean latency, GOF value, intensity, and location and orientation of MNI coordinates of each ECD are shown in Table 1.

**Table 1:** Properties of the equivalent current dipoles in the insular and the posterior cingulate cortices related to listening to the metronome after conditioning

	**Insular cortex**		**Posterior cingulate cortex**
	**Right**	**Left**	
No. of participants	4	4	4
Peak latency (ms)	191.0 ± 10.6	184.0 ± 4.6	468.0 ± 13.5
GOF (%)	95.3 ± 2.1	93.5 ± 1.6	95.8 ± 1.5
Intensity (nA · m)	25.0 ± 2.8	16.7 ± 2.5	16.4 ± 3.3
Locations*			
x (mm)	41.8 ± 2.7	−41.7 ± 3.1	−8.3 ± 2.6
y (mm)	−20.8 ± 4.2	−26.2 ± 6.1	−47.3 ± 5.0
z (mm)	8.9 ± 4.2	3.3 ± 7.9	25.7 ± 6.4
Orientations*			
θ (degrees)	43.6 ± 12.9	34.8 ± 5.7	116.5 ± 23.3
φ (degrees)	82.6 ± 9.3	99.7 ± 12.9	275.6 ± 56.1

Data are shown as mean ± SEM (n = 8). *GOF* goodness of fit.

*Converted to Montreal Neurological Institute coordinates.

## Discussion

Our results demonstrated that mental fatigue sensation was induced by the metronome sounds which had been associated with mental fatigue sensation. In addition, we found that the insular cortex is involved in the neural substrate. The involvement of the PCC in the neural substrate was also suggested. Our results support the idea that classical conditioning of the fatigue sensation can take place in humans, and this conditioning contributes to the chronic fatigue sensation as proposed by the Co-conditioning Theory of Fatigue [1].

Based on our previous findings that performing two-back task trials for 30 min induces mental fatigue and mental fatigue sensation [11,12], the metronome sounds were started 30 min after the start of the conditioning session. The level of mental fatigue sensation was increased after the two-back task trials for 60 min (Figure 2B). Fatigue sensation was caused by listening to the metronome sounds in the second MEG session, and the level of this fatigue sensation was significantly higher than that caused in the first MEG session (Figure 2C). In addition, there was a positive correlation between the level of the fatigue sensation caused by the metronome sounds and the notice level of the sounds during the conditioning session (Figure 3). Importantly, it was confirmed that repetitive listening to metronome sounds (i.e., participants listened to the metronome sounds for six min but did not perform conditioning session on the first day and listened to the sounds again on the second day) did not induce the fatigue sensation. These observations show that mental fatigue sensation caused on the second day was due to the conditioning session performed on the first day, suggesting that classical conditioning of the fatigue sensation took place in our experiment. Interestingly, the LF/HF ratio after the second MEG session showed a tendency toward increase compared to that before the session. It seems that the increase in the LF and the decrease in the HF resulted in an increase in the LF/HF ratio in the second MEG session (Figure 4). Since it has been consistently shown that fatigue-inducing mental load reduces vagal nerve activities, that is, the decrease in the HF [21-26], resulting increase in the LF/HF ratio are a characteristic feature of fatigue. In fact, it has been reported that increases in the LF/HF ratio and decreases in HF were associated with mental fatigue in healthy participants [11,13] and an increase in the LF/HF ratio has been observed in patients with chronic fatigue syndrome [27,28]. Taken together, not only fatigue sensation but also changes in an objective indicator of fatigue can be induced by conditioning of the fatigue sensation.

In our study, activation in the insular cortex was related to the mental fatigue sensation evoked through conditioning of the fatigue sensation. Because the acquisition of conditioning took place after the first MEG session, the neural activities observed only in the second MEG session were considered to be related to the acquisition of conditioning of the mental fatigue sensation [29]. The activation in the insular cortex observed in our study may be related to the aversive value regarding the fatigue sensation induced by the conditioning procedure [30]. A number of investigations have shown that the insular cortex is activated when aversive stimuli are presented. For example, inhalation of odorants producing a strong feeling of disgust caused activation in the insular cortex [31], nausea produced by visually induced circular vection resulted in activation in the insular cortex [32], and observing aversive pictures also activated the insular cortex [33]. The insular cortex is involved in monitoring the state of the body (i.e., sensing the physiological condition of the entire body) [34,35] and sustained responsiveness to external stimuli [36]. Since the fatigue sensation is an unpleasant sensation accompanying fatigue, the fatigue sensation is aversive by nature. Furthermore, since the fatigue sensation is one of the biological alarms used by the body to avoid upsetting homeostasis, monitoring information about fatigue as a noxious stimulus is of value. In fact, one study showed that activation in the insular cortex was positively correlated to the level of fatigue sensation induced by the typhoid vaccination [37]. Therefore, the aversive value related to the fatigue sensation induced by the metronome sounds after the conditioning session would have caused activation in the insular cortex.

There are limitations to our study: First, sitting for 60 min and listening to the metronome sounds for 30 min were not included in the control group. Since sitting for 60 min and listening to the metronome sounds for 30 min might cause fatigue sensation even if fatigue-inducing task trials were not performed, we did not include them in the control group. However, it is likely that repeated pairing of the metronome sounds with mental fatigue sensation in the first day, rather than sitting for 60 min and listening to the metronome sounds for 30 min, may induce fatigue sensation in the second day, because we confirmed that repetitive listening to the metronome sounds did not induce fatigue sensation. Second, pre-exposure to the conditioned and unconditioned stimuli were included in our experiment. Although the pre-exposure of the conditioned and unconditioned stimuli may have weakened the conditioning effect, we observed that the fatigue sensation was induced by listening to the metronome sounds after the conditioning session, suggesting that the participants successfully acquired the conditioned response during conditioning session. Further studies are needed to conclude that the metronome sounds induced mental fatigue sensation through classical conditioning. Third, the subjective level of mental fatigue immediately before the MEG session in the second day was lower compared to that in the first day. However, since the increase of the fatigue sensation in the second MEG session was greater than that in the first MEG session (Figure 2A), the fatigue sensation caused by listening to the metronome sounds in the second MEG session seems to be due to the condition procedure performed in the first day (Figure 2C). Fourth, neural activities during the acquisition phase were not assessed in our study. It was difficult to examine neural activities during fatigue-inducing mental task trials because movements of the participants’ hands to press buttons caused electromagnetic noises during MEG measurements. Therefore, we adopted a study design comparing neural activities after the conditioning session with those before the conditioning session. Fifth, a limited number of participants were tested. To generalize our results, studies involving a larger numbers of participants are needed.

## Conclusions

We demonstrated that the metronome sounds caused mental fatigue sensation as a result of repeated pairings of the sounds with mental fatigue. This means that the expectation of fatigue can cause fatigue sensation as suggested by co-conditioning theory. We also showed that the insular cortex is involved in the neural substrates of this phenomenon. Our findings may help clarify the neural mechanisms of the fatigue sensation as well as aid in the development of the treatment methods for patients suffering from severe fatigue sensation such as chronic fatigue syndrome.

## Abbreviations

MEG: Magnetoencephalography; ECD: Equivalent current dipole; VAS: Visual analogue scale; GOF: Goodness of fit; MRI: Magnetic resonance imaging; ECG: Electrocardiography; LF: Low-frequency power; HF: High-frequency power; PCC: Posterior cingulate cortex; ACC: Anterior cingulate cortex; PET: Positron emission tomography.

## Competing interests

We declare that we have no competing interests.

## Authors’ contributions

AI took part in planning and designing the experiment, collected the data, performed the data analyses and drafted the manuscript. MT took part in planning and designing the experiment, performed the data analyses and helped drafting the manuscript. MI, CK, and EY took part in planning and designing the experiment, collected the data, and performed the data analyses. YW took part in the planning and designing the experiment and helped drafting the manuscript. All the authors read and approved the final manuscript.
